# Structured Moderate Exercise and Biomarkers of Kidney Health in Sedentary Older Adults: The Lifestyle Interventions and Independence for Elders Randomized Clinical Trial

**DOI:** 10.1016/j.xkme.2023.100721

**Published:** 2023-09-13

**Authors:** Anoop Sheshadri, Mason Lai, Fang-Chi Hsu, Scott R. Bauer, Shyh-Huei Chen, Warren Tse, Vasantha Jotwani, Gregory J. Tranah, Jennifer C. Lai, Stein Hallan, Roger A. Fielding, Christine Liu, Joachim H. Ix, Steven G. Coca, Michael G. Shlipak

**Affiliations:** 1 Department of Medicine, University of California San Francisco, San Francisco, CA; 2 Department of Medicine, San Francisco VA Health Care System, San Francisco, CA; 3 Department of Biostatistics and Data Science, Wake Forest University School of Medicine, Winston-Salem, NC; 4 California Pacific Medical Center, San Francisco, CA, United States; 5 Department of Clinical and Molecular Medicine, Norwegian University of Science and Technology, Trondheim, Norway; 6 Jean Mayer USDA Human Nutrition Research Center on Aging at Tufts University, Boston, MA; 7 Department of Medicine, Stanford University School of Medicine, Stanford, CA; 8 Geriatric Research Education and Clinical Center, Palo Alto VA Health Care System, Palo Alto, CA; 9 Department of Medicine, University of California San Diego, La Jolla, CA; 10 Department of Internal Medicine, Icahn School of Medicine at Mount Sinai, New York, NY

**Keywords:** Biomarkers, kidney health, exercise, physical activity, older adults

## Abstract

**Rationale & Objective:**

In the Lifestyle Interventions and Independence for Elders (LIFE) trial, a structured exercise intervention slowed kidney function decline in sedentary older adults. Biomarkers of kidney health could distinguish potential mechanisms for this beneficial effect.

**Study Design:**

Randomized controlled trial.

**Setting & Population:**

A total of 1,381 sedentary adults aged 70-89 years enrolled in the LIFE trial.

**Intervention:**

Structured, 2-year, moderate-intensity exercise intervention versus health education.

**Outcomes:**

Physical activity was measured by step count. Primary outcomes were changes in 14 serum and urine biomarkers of kidney health collected at baseline, year 1, and year 2. We determined the effect of randomization on changes in kidney measures and then evaluated observational associations of achieved activity on each measure.

**Results:**

Participants assigned to exercise walked on average 291 more steps per day than participants assigned to health education. The intervention was not significantly associated with changes in biomarkers of kidney health. In observational analyses, persons in the highest versus lowest quartile of activity (≥3,470 vs <1,568 steps/day) had significant improvement in urine albumin (mean, −0.22 mg albumin/g urine creatinine [interquartile range (IQR), −0.37 to −0.06]), alpha-1-microglobulin (−0.18 mg/L [−0.28 to −0.08]), trefoil factor-3 (−0.24 pg/mL [−0.35 to −0.13]), epidermal growth factor (0.19 pg/mL [0.06-0.32]), uromodulin (0.06 pg/mL [0.00-0.12]), interleukin 18 (−0.09 pg/mL [−0.15 to −0.03]), neutrophil gelatinase-associated lipocalin (−0.16 pg/mL [−0.24 to −0.07]), monocyte chemoattractant protein-1 (−0.25 pg/mL [−0.36 to −0.14]), clusterin (-0.16 pg/mL [−0.30 to −0.02]), serum tumor necrosis factor receptor-1 (−0.25 mg/dL [−0.39 to −0.11]) and tumor necrosis factor receptor-2 (−0.30 mg/dL [−0.44 to −0.16]). In sensitivity analyses, incremental changes in activity were most impactful on urine interleukin 18 and serum tumor necrosis factor-1.

**Limitations:**

The original study was not designed to assess the impact on kidney health. Non-white individuals and patients with advanced chronic kidney disease are underrepresented.

**Conclusions:**

Randomization to structured exercise did not improve kidney health at a group level. However, higher exercise was associated with concurrent improvements in biomarkers of glomerular injury, tubular function/repair, tubular injury, generalized inflammation, and tubulointerstitial repair/fibrosis.

**Plain-Language Summary:**

In the Lifestyle Interventions For Elders (LIFE) study, randomization to an exercise and physical activity intervention improved the slope of estimated glomerular filtration rate over 2 years compared with health education among older adults. In this study, we sought to determine whether there were specific biomarkers of kidney health that were affected by the exercise and physical activity intervention to investigate potential mechanisms for this positive impact on kidney decline. We found that randomization to the intervention did not improve any of the 14 measures of kidney tubule health. However, in observational analyses, higher activity was independently associated with improvements in several domains, especially tubular injury and generalized inflammation. These results help to clarify the impact of physical activity on kidney health.

Recent trials indicate that physical activity is beneficial in elderly patients with chronic kidney disease (CKD)^1^ and that high-intensity exercise can transiently improve kidney health in patients with moderate CKD^2^; however, apart from kidney filtration, it is not known which domains of kidney health are improved by exercise. Although it is known that estimated glomerular filtration rate (eGFR) declines with age, the pathological processes of age-related kidney disease likely begin decades before CKD clinical criteria are fulfilled.^3^ Tubulointerstitial disease is a hallmark of nephrosclerosis, a histologic pattern of the aging kidney that includes glomerular sclerosis and reductions in nephron size and number.^4^ Tubulointerstitial disease is a prominent component of age-related kidney disease that remains largely unevaluated in clinical practice and research. In recent years, novel urine and blood biomarkers have been developed that can improve our assessment of kidney health.

Biomarkers of kidney health can be loosely classified as representing glomerular damage, glomerular function, tubular injury, tubular dysfunction, and tubulointerstitial repair/fibrosis.^5^ In numerous cohorts, higher levels of biomarkers reflecting kidney injury and dysfunction have been associated with increased risks of mortality, cardiovascular disease, and rapid eGFR decline.^6^, ^7^, ^8^, ^9^, ^10^, ^11^, ^12^ Similarly, urine biomarkers representing the capacity for regeneration or repair, such as uromodulin (UMOD) and epidermal growth factor (EGF), have been shown to be associated with lower risks of mortality, CKD risk, and cardiovascular disease.^12^, ^13^, ^14^

The Lifestyle Interventions and Independence for Elders (LIFE) trial was a multicenter trial designed to test the effect of a combined exercise and activity intervention on major mobility and disability in community-dwelling, sedentary older adults.^15^ In a LIFE ancillary study, our group recently demonstrated that this intervention was associated with slower decline in the estimated glomerular filtration rate by cystatin C (eGFR_CysC_). Furthermore, those who achieved higher activity levels derived greater benefits to eGFR_CysC_ preservation from the intervention. Crucially, the association of higher activity with slower eGFR decline was independent of changes in blood pressure or body weight, suggesting that physical activity can directly mitigate the decline in glomerular function in older adults.

The present study aimed to explore the following: 1) the effects of randomization to an exercise and activity intervention on domains of kidney health beyond glomerular function and 2) the associations of physical activity as measured by step counts with these domains of kidney health. To achieve these objectives, we used urine and serum specimens that were stored at the baseline, year 1 and year 2 visits of the LIFE trial to measure multiple biomarkers reflecting various domains of kidney health. We hypothesized that both randomization to the exercise intervention and higher step counts during the LIFE trial would be associated with relative improvements in biomarkers reflecting glomerular injury, tubular function and repair, tubular injury, generalized inflammation, and tubulointerstitial repair/fibrosis.

## Methods

### Study Design and Patients

The LIFE trial was a phase 3 multicenter, randomized controlled trial of a moderate-intensity physical activity and exercise program compared with a “successful aging” health education program, involving 1,635 sedentary older adults enrolled between February 2010 and December 2013 across 8 centers in the United States.^15^ The LIFE trial included men and women aged 70-89 years with the following characteristics: 1) sedentary lifestyle, defined by reporting <20 minutes per day of regular physical activity and <125 minutes per week of moderate physical activity; 2) at high risk of disability based on a score of <10 (but greater than 4) on the Short Physical Performance Battery; 3) ability to complete the 400-m walk test without an assistive device; 4) absence of cognitive impairment defined by a score of >80 on the Modified Mini-Mental State Examination; and 5) willingness to consent to randomization. Exclusion criteria included unstable chronic disease, factors that would likely affect adherence to the intervention, or underlying conditions that might limit survival to study completion. There were no specific exclusions related to nondialysis CKD, but patients who were receiving dialysis at baseline were excluded.

All participants in the LIFE study underwent informed consent before participation. This study was approved by the University of California, San Francisco Institutional Review Board (18-25182).

### Interventions

Participants in the LIFE trial underwent block randomization stratified by field center and sex. The intervention arm underwent a combined-activity and functional exercise intervention. The activity component targeted 150 minutes of moderate-intensity exercise per week, primarily composed of walking, and the functional exercise component included strength, flexibility, and balance training. Participants were expected to attend exercise sessions at their field center twice weekly and to conduct home-based activities 3-4 times weekly throughout the trial duration. Exercise sessions were individualized, and participants were expected to progress toward a goal of 30 minutes of daily walking, 10 minutes of lower extremity strength training, 10 minutes of balance training, and large muscle flexibility exercises. Intensity started low and increased over the first weeks of the intervention. Participants were asked to walk at a self-perceived exertion of 13 (“somewhat hard”) on the original Borg scale and perform lower extremity strength exercises at a self-perceived exertion of 15-16 (“hard”). The health education arm of the trial involved weekly workshops over the first 26 weeks followed by monthly workshops. These sessions addressed a variety of health topics relevant to older adults (eg, how to effectively negotiate the health care system, how to travel safely, recommended preventive services and screenings at different ages, and where to go for reliable health information, nutrition) but did not specifically address physical activity.^15^

### Measurements

Baseline assessments conducted before randomization included self-reported socio-demographic information (age, sex, self-reported race, and education), medical history (presence of diabetes, hypertension, and cardiovascular disease), and physical examination (body mass index and systolic blood pressure). Physical activity was assessed using accelerometers (ActiGraph wGT3X-BT) at baseline and at 6, 12, and 24 months of follow-up; on each occasion participants wore the accelerometer on their hip for 7 days. Only data collected at baseline and 12 and 24 months were used for these analyses because they were contiguous with the assessments of biomarkers of kidney health. Details of these measures were described elsewhere.^15^^,^^16^ Accelerometers reported the total step counts per day. All accelerometer data were adjusted for wear time.

During the trial, serum and urine specimens were collected at the baseline, year 1 and year 2 visits and immediately stored at −80 °C. Urine alpha-1 microglobulin (A1M) and albumin were measured using the Siemens BN II Nephelometer. Urine creatinine level was measured with a commercially available colorimetric kit using the Jaffe reaction from R&D Systems. Urine biomarkers (trefoil factor 3 [TFF3], epidermal growth factor [EGF], UMOD, interleukin 18 [IL-18], neutrophil gelatinase-associated lipcalin [NGAL], kidney injury molecule 1 [KIM1], monocyte chemoattractant protein 1 [MCP1], clusterin, and Chitinase-3-like protein 1 [YKL40]) were measured using multiplex assay kits from Meso Scale Discovery (MSD) according to manufacturer protocols. Serum markers (KIM1, TNFR1, and TNFR2) were also measured using a multiplex plate from Meso Scale Discovery (MSD). For each biomarker, all measures for an individual participant were conducted concurrently to avoid assay drift.

### Statistical Analyses

Baseline characteristics were summarized by randomization groups using means and standard deviations (SDs) for continuous variables or counts and percentages for discrete variables. Here, *t* tests, Wilcoxon rank sum tests, and χ^2^ tests were used to compare normally distributed, nonnormally distributed, and discrete characteristics, respectively, between participants with and without accelerometry or biomarker measures. The associations of the intervention on the outcomes of standardized changes in biomarkers of kidney health were analyzed using the mixed effects models with an unstructured covariance matrix. Urine biomarkers were indexed to 1 g of urine creatinine. We standardized the changes in biomarkers to compare the intervention effects across different outcomes qualitatively. The interpretation of the intervention effect was based on the standardized change in biomarker (unit: SD) when comparing physical activity versus health education. Covariates included baseline biomarker, sex and field center (stratified variables), intervention, clinic visit (year 1 and year 2), and intervention-by-visit interaction. Hypothesis tests for intervention effects at the year 1 and year 2 assessment visits were performed using contrasts of the year 1 and year 2 intervention means. Overall comparisons between intervention groups across follow-up visits were obtained using a contrast to compare average effects across both follow-up visits.

We next evaluated associations between measured time-updated step count during follow-up with standardized changes in biomarkers of kidney health. The associations between standardized time-updated step counts with standardized changes in biomarkers of kidney health function were determined using mixed effects models. The covariates in the basic model included baseline biomarker, sex and field center (stratified variables), intervention, clinic visit, and intervention-by-visit interaction. Because these analyses were not comparing randomized groups, we additionally adjusted for potential confounding variables, including age, body mass index, systolic blood pressure, race, education, diabetes, cardiovascular disease, and hypertension. All analyses of urine biomarkers were conducted using the biomarkers indexed for urine creatinine. Step count was also analyzed using quartiles for ease of comparing associations with standardized levels of the biomarkers; high versus low quartiles of activity were compared across the intervention period to ascertain the cumulative effect of activity on biomarkers of kidney health.

In our prior study,^17^ we also examined the association of time spent in moderate-intensity activity on changes in kidney function. However, there was considerable overlap between time spent in moderate-intensity activity and step count, and step count appeared to best capture the relationship between activity and kidney function. Therefore, we also used step count as our primary predictor for these analyses of biomarkers of kidney health.

We next attempted to distinguish associations of incremental changes from baseline in activity, as opposed to cumulative effect of activity over 2 years, with parallel changes in the biomarkers of kidney health across the entire cohort. In these analyses, the baseline step count and the changes in step count from baseline were separate covariates for the outcomes of longitudinal biomarkers of kidney health after adjustment for baseline biomarker, intervention, sex, clinical sites, age, body mass index, time-varying systolic blood pressure, race, education, diabetes, cardiovascular disease, hypertension, visit, and intervention-by-visit interaction. Both baseline step count and outcome measures (ie, changes in biomarkers) are standardized. Change in step count was calculated as standardized follow-up step count minus standardized baseline step count.

For all analyses, a 2-sided *P* value of <0.05 was considered to indicate statistical significance. We did not adjust for multiple comparisons because all of the outcomes are interrelated as manifestations of kidney tubule health; our global hypothesis was that physical activity would be associated with improvements in kidney damage and dysfunction. The statistical software used to conduct these analyses included SAS version 9.4 and R version 3.6.1.

## Results

### Study Cohort

Among the 1,635 participants of the LIFE study, 1,381 had available specimens for biomarkers of kidney health (Figure S1). Participants without available specimens for biomarkers were more likely to be women, have a lower prevalence of diabetes, and walked less at baseline than those with available specimens (Table S1). Participants with and without accelerometry measures were similar (Table S2). The average age of included participants was 79 ± 5 years, and two-thirds were women. Here, 17% of participants self-reported as Black, 76% as White, and 7% as other. The mean eGFR_CysC_ at baseline was 54 ± 17 mL/min/1.73 m^2^ (Table 1) and eGFR values ranged from 11-109 mL/min/1.73 m^2^. Diabetes was present in 29% of participants, hypertension in 72%, and cardiovascular disease in 30%. In addition, 66% of participants had an eGFR_CysC_ < 60 mL/min/1.73 m^2^. There were no significant differences between those randomized to the intervention or health education. At baseline, the step counts for participants in the intervention arm and control arm were 2,688 ± 1,377 steps and 2,743 ± 1,586 steps, respectively. At years 1 and 2, those in the intervention arm had recorded step counts that were on average 19% higher than the control arm.

**Table 1:** Baseline Characteristics of Participants in the LIFE Trial Stratified by Randomization Arm: Physical Activity Intervention versus Health Education

	PA (n = 686)	HE (n = 695)	Overall (n = 1,381)
Age, y, mean (SD)	78.6 (5.2)	79.1 (5.3)	78.8 (5.2)
Female, n (%)	447 (65.2)	460 (66.2)	907 (65.7)
Race, n (%)			
Black	137 (20.0)	103 (14.8)	240 (17.4)
White	508 (74.1)	543 (78.1)	1051 (76.1)
Other	41 (6.0)	49 (7.1)	90 (6.5)
Education, n (%)			
≤ High school	234 (34.2)	229 (33.1)	463 (33.7)
College	284 (41.5)	286 (41.3)	570 (41.4)
Postgraduate	166 (24.3)	177 (25.6)	343 (24.9)
BMI (kg/m^2^), mean (SD)	30.2 (5.7)	30.3 (6.0)	30.2 (5.9)
Diabetes status, n (%)			
No diabetes	327(47.7)	344 (49.5)	671 (48.6)
Impaired fasting glucose	170 (24.84)	147 (21.2)	317 (23.0)
Diabetes	189 (27.6)	204 (29.4)	393 (28.5)
CVD, n (%)	202 (29.5)	214 (30.8)	416 (30.1)
Hypertension, n (%)	484 (70.8)	503 (72.8)	987 (71.8)
SBP (mmHg), mean (SD)	127.7 (18.3)	127.1 (17.6)	127.4 (18.0)
eGFR_CysC_ (mL/min/1.73 m^2^), mean (SD)	53.9 (17.5)	53.6 (17.2)	53.8 (17.3)
CKD (eGFR_CysC_ < 60), n (%)^a^	368 (65.8)	382 (66.0)	750 (65.9)

Abbreviations: LIFE, Lifestyle Interventions and Independence for Elders; PA, physical activity; HE, health education; BMI, body mass index; CVD, cardiovascular disease; SBP, systolic blood pressure; eGFR_CysC_, estimated glomerular filtration rate by cystatin C.

a 243 participants lacked eGFR_CysC_ data (N for eGFR_CysC_ = 1,138).

### Effect of Randomization to Physical Exercise on Biomarkers of Kidney Health

Randomization to the structured physical activity and exercise intervention over 2 years did not result in significant changes in any of the domains of kidney health, including measures of glomerular injury, tubular function and repair, tubular injury, generalized inflammation, and tubulointerstitial repair/fibrosis (Table 2). When examining the intervention effect at each follow-up year, only TFF3 shows a negative association at year 2 (beta estimate = −0.10, *P* value = 0.02) (Table S3).

**Table 2:** Effect of Randomization to Physical Activity Intervention Versus Health Education Control on Biomarkers of Tubular Function and Injury Over the Study Period

Biomarker (SD)	Standardized Change in Biomarker (95% CI)	*P* Value
**Glomerular Injury**		
Urine albumin (3.47)^a^	−0.01 (−0.11 to 0.09)	0.89
**Tubular Function and Repair**		
A1M (0.54)^b^	0.002 (−0.08 to 0.08)	0.96
TFF3 (18.9)^c^	−0.05 (−0.13 to 0.02)	0.16
EGF (22.2)^c^^,^^d^	−0.04 (−0.13 to 0.05)	0.41
UMOD (219129)^c^^,^^d^	−0.01 (−0.05 to 0.02)	0.45
**Tubular Injury**		
IL-18 (0.73)^c^	0.0009 (−0.04 to 0.04)	0.96
NGAL (6446)^c^	−0.07 (−0.17 to 0.02)	0.14
KIM1 (urine) (16.9)^c^	0.04 (−0.05 to 0.12)	0.43
KIM1 (serum) (189)	0.04 (−0.06 to 0.15)	0.44
**Generalized Inflammation**		
TNFR1 (serum) (1,090)	−0.02 (−0.12 to 0.09)	0.77
TNFR2 (serum) (3,898)	0.08 (−0.12 to 0.18)	0.12
**Tubulointerstitial Repair/Fibrosis**		
MCP1 (3.09)^c^	−0.02 (−0.09 to 0.06)	0.68
Clusterin (6,376)^c^	−0.03 (−0.12 to 0.06)	0.53
YKL40 (219.3)^c^	−0.04 (−0.12 to 0.05)	0.38

*Note:* Adjusted for baseline biomarker, intervention, visit, sex, clinical site, and interaction between visit and intervention. All urine biomarkers are indexed to Cr (ie, biomarker/Cr). Changes in biomarkers are standardized.

Abbreviations: A1M, alpha-1 microglobulin; CI, confidence interval; EGF, epidermal growth factor; IL, interleukin; KIM1, kidney injury molecule 1; MCP1, monocyte chemoattractant protein 1; NGAL, neutrophil gelatinase-associated lipcalin; SD, standard deviation; TFF3, trefoil factor 3; UMOD, uromodulin; YKL40, Chitinase-3-like protein 1.

a mg Alb/g Cr.

b mg/L per mg/dL of UCr.

c pg/mL per mg/dL of UCr.

d Higher levels of EGF and UMOD reflect improved kidney health. Lower levels of all other biomarkers reflect improved kidney health or improved systemic inflammation.

### Associations of Time-Updated Step Count With Standardized Biomarkers of Kidney Health

**Figure 1:**
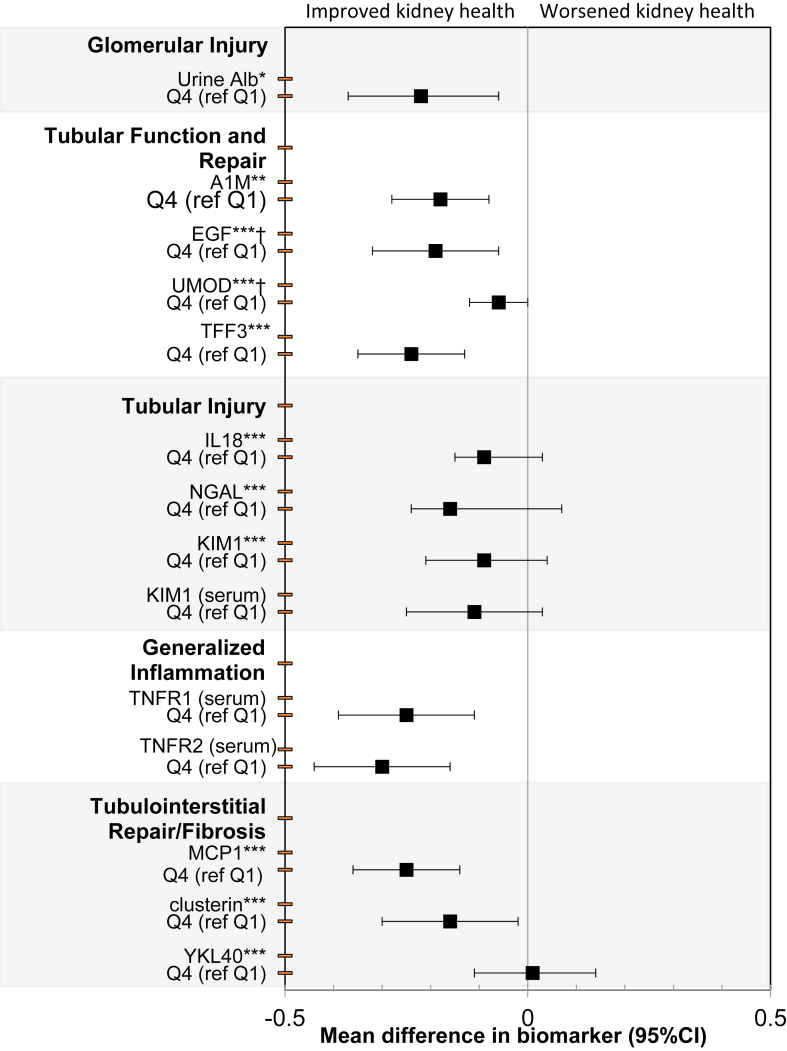
Associations of time-updated step count (high vs low quartile) with changes in 14 biomarkers of kidney health (scaled/SD) using mixed effects models in the LIFE trial. All models are adjusted for intervention, sex, clinical sites, age, BMI, time-updated SBP, race, education, diabetes, CVD, hypertension, visit, and intervention-by-visit interaction. Note: All urine biomarkers are indexed to urine Cr. Abbreviations: LIFE, Lifestyle Interventions and Independence for Elders; BMI, body mass index; SBP, systolic blood pressure; CVD, cardiovascular disease. †BP, systolic blood pressure; CVD, cardiovascular diseaseites, age, BMI, time-updated SBP, race, education, diabetes, CVD, hypertension, visit, and interventionHealth ABC) Studythors & Journmproved kidney health or systemic inflammation. ∗ mg Alb/g Cr ∗∗ mg/L per mg/dL of UCr ∗∗∗pg/mL per mg/dL of UCr.

In contrast, when time-updated step count was evaluated comparing high versus low quartiles of activity (Fig 1) or as a linear predictor (Table 3), greater amounts of activity were significantly associated with improvements in biomarkers. Specifically, in the analysis by quartiles, a higher time-updated step count was associated with improvements in markers of glomerular injury (urinary albumin), tubular function and repair (A1M, TFF3, EGF, and UMOD), tubular injury (IL-18 and NGAL), inflammation (TNFR1 and TNFR2), and tubulointerstitial repair/fibrosis (MCP1 and clusterin). When time-updated step count was analyzed as a linear measure, participants with greater activity had similar improvements in measures of kidney health; these associations were also observed in the analyses by quartiles of step count but not for EGF, UMOD, and clusterin. A higher time-updated step count was not associated with improved levels of urine or serum KIM1 or urine YKL40 in either the quartile or linear analyses.

**Table 3:** Associations of Time-Updated Step Count With Repeated Measures of Kidney Tubule Biomarkers^a^ in the LIFE Study

Standardized Change in Biomarkers (Beta coefficients; *P* Value)				
Per SD of steps (±1487)	Q1 (139-1,568, N = 236)	Q2 (1,568-2,362, N = 279)	Q3 (2,362-3,470, N = 298)	Q4 (3,470-13,598, N = 313)
**Glomerular Injury**				
Urine albumin^b^				
−0.16 (−0.30 to −0.03) *P* = 0.03	Ref.	−0.16 (−0.30 to −0.03); *P* = 0.02	−0.04 (−0.19 to 0.10); *P* = 0.54	−0.22 (−0.37 to -0.06) *P* < 0.01
**Tubular Function and Repair**				
A1M^c^				
−0.03 (−0.07 to −0.01); *P* = 0.02	Ref.	−0.14 (−0.23 to −0.05); *P* < 0.01	−0.12 (−0.21 to −0.02); *P* = 0.01	−0.18 (−0.28 to −0.08) *P* < 0.01
TFF3^d^				
−0.06 (−0.09 to −0.02); *P* < 0.01	Ref.	−0.14 (−0.23 to −0.05); *P* < 0.01	−0.19 (−0.29 to −0.09); *P* < 0.01	−0.24 (−0.35 to −0.13) *P* < 0.01
EGF^d^^,^^e^				
0.03 (−0.01 to 0.07); *P* = 0.17	Ref.	0.02 (−0.10 to 0.13); *P* = 0.79	0.08 (−0.04 to 0.20); *P* = 0.17	0.19 (0.06-0.32) *P* < 0.01
UMOD^d^^,^^e^				
0.01 (0.00-0.03); *P* = 0.10	Ref.	0.04 (−0.01 to 0.09); *P* = 0.14	0.06 (0.00-0.11); *P* = 0.04	0.06 (0.00-0.12) *P* = 0.03
**Tubular Injury**				
IL-18^d^				
−0.04 (−0.06 to −0.02); *P* < 0.01	Ref.	−0.06 (−0.11 to 0.00); *P* = 0.05	−0.06 (−0.12 to −0.06); *P* = 0.03	−0.09 (−0.15 to −0.03) *P* < 0.01
NGAL^d^				
−0.04 (−0.07 to −0.01); *P* < 0.01	Ref.	−0.07 (−0.16 to 0.02); *P* = 0.11	−0.08 (−0.16 to 0.01); *P* = 0.09	−0.16 (−0.24 to −0.07) *P* < 0.01
KIM1^d^				
−0.02 (−0.06 to 0.02); *P* = 0.26	Ref.	−0.09 (−0.20 to 0.03); *P* = 0.14	−0.04 (−0.16 to 0.08); *P* = 0.48	−0.09 (−0.21 to 0.04) *P* = 0.19
KIM1 (serum)				
−0.01 (−0.05 to 0.02); *P* = 0.54	Ref.	−0.08 (−0.20 to 0.03); *P* = 0.16	−0.10 (−0.22 to 0.03); *P* = 0.12	−0.11 (−0.25 to 0.03); *P* = 0.12
**Generalized Inflammation**				
TNFR1 (serum)				
−0.06 (−0.10 to −0.02); *P* < 0.01	Ref.	−0.17 (−0.29 to −0.06); *P* < 0.01	−0.23 (−0.36 to −0.11); *P* < 0.01	−0.25 (−0.39 to −0.11) *P* < 0.01
TNFR2 (serum)				
−0.07 (−0.11 to −0.03); *P* < 0.01	Ref.	−0.18 (−0.31 to −0.06); *P* < 0.01	−0.24 (−0.37 to −0.11); *P* < 0.01	−0.30 (−0.44 to −0.16) *P* < 0.01
**Tubulointerstitial Repair/Fibrosis**				
MCP1^d^				
−0.05 (−0.09 to −0.02); *P* < 0.01	Ref.	−0.11 (−0.21 to −0.01) *P* = 0.02	−0.14 (−0.25 to −0.04); *P* < 0.01	−0.25 (−0.36 to −0.14) *P* < 0.01
Clusterin^d^				
−0.03 (−0.08 to 0.01); *P* = 0.12	Ref.	−0.07 (−0.06 to 0.05); *P* = 0.26	−0.12 (−0.25 to 0.01); *P* = 0.07	−0.16 (−0.30, to −0.02) *P* = 0.02
YKL40^d^				
0.00 (−0.04 to 0.04); *P* = 0.92	Ref.	0.05 (−0.06 to 0.16); *P* = 0.37	0.05 (−0.06 to 0.17); *P* = 0.36	0.01 (−0.11 to 0.14) *P* = 0.84

*Note:* All models adjusted for intervention, sex, clinical sites, age, BMI, time-varying SBP, race, education, diabetes, CVD < hypertension, visit, and intervention-by-visit interaction. All urine biomarkers are indexed to Cr (ie, biomarker/Cr).

Abbreviations: A1M, alpha-1 microglobulin; CI, confidence interval; EGF, epidermal growth factor; IL, interleukin; KIM1, kidney injury molecule 1; MCP1, monocyte chemoattractant protein 1; NGAL, neutrophil gelatinase-associated lipcalin; SD, standard deviation; TFF3, trefoil factor 3; UMOD, uromodulin; YKL40, Chitinase-3-like protein 1.

a Changes in biomarkers are standardized. Results expressed as proportions of standard deviation for change in each biomarker. Primary covariate of interest is the step count at each follow-up visit (a time-varying variable).

b mg Alb/g UCr.

c mg/L per mg/dL of UCr.

d pg/mL per mg/dL of UCr.

e Higher levels of EGF and UMOD reflect improved kidney health. Lower levels of all other biomarkers reflect improved kidney health or improved systemic inflammation.

### Associations of Incremental Change in Step Count with Biomarkers of Kidney Health

Incremental changes in step count from baseline demonstrated significant associations with improvements in tubular injury (IL-18) and generalized inflammation (TNFR1). The coefficients of all remaining biomarkers except for serum KIM1 were directionally associated with improvements in kidney health. Effect sizes of incremental changes in step count were somewhat smaller than the associations with baseline step count (Table 4).

**Table 4:** Associations of Baseline Step Count and Incremental Change in Step Count From Baseline With Standardized Change in Biomarkers in the LIFE Study

Standardized Change in Biomarkers (Beta coefficients; *P* Value)	
Baseline Steps (per SD)	Incremental Change in Standardized Steps
**Glomerular Injury**	
Urine albumin^a^	
−0.02 (−0.07 to 0.02); *P* = 0.35	−0.006 (−0.04 to 0.03); *P* = 0.74
**Tubular Function and Repair**	
A1M^b^	
−0.03 (−0.06 to −0.01); *P* = 0.01	−0.03 (−0.06 to 0.00); *P* = 0.07
TFF3^c^	
−0.07 (−0.11 to −0.03); *P* < 0.01	−0.02 (−0.07 to 0.02); *P* = 0.25
EGF^c^^,^^d^	
0.04 (−0.01 to 0.10); *P* = 0.13	0.01 (−0.04 to 0.07); *P* = 0.59
UMOD^c^^,^^d^	
0.02 (0.00-0.04); *P* = 0.03	0.01 (−0.02 to 0.03); *P* = 0.55
**Tubular Injury**	
IL−18^c^	
−0.01 (−0.03 to 0.01); *P* = 0.29	−0.04 (−0.07 to −0.02); *P* < 0.01
NGAL^c^	
−0.06 (−0.11 to −0.01); *P* = 0.02	−0.03 (−0.07 to 0.01); *P* = 0.19
KIM1^c^	
−0.03 (−0.07 to 0.02); *P* = 0.23	−0.02 (−0.07 to 0.04); *P* = 0.54
KIM1 (serum)	
−0.02 (−0.07 to 0.03); *P* = 0.49	0.00 (−0.04 to 0.05); *P* = 0.91
**Generalized Inflammation**	
TNFR1 (serum)	
−0.07 (−0.13 to −0.01); *P* = 0.02	−0.05 (−0.11 to 0.00); *P* = 0.05
TNFR2 (serum)	
−0.08 (−0.14 to −0.03); *P* < 0.01	−0.05 (−0.10 to 0.01); *P* = 0.09
**Tubulointerstitial Repair/Fibrosis**	
MCP1^c^	
−0.05 (−0.09 to −0.02); *P* < 0.01	−0.03 (−0.07 to 0.01); *P* = 0.09
Clusterin^c^	
−0.03 (−0.08 to 0.02); *P* = 0.22	−0.02 (−0.08 to 0.03); *P* = 0.35
YKL40^c^	
−0.02 (−0.04 to 0.01); *P* = 0.17	−0.01 (−0.03 to 0.01); *P* = 0.49

*Note:* Changes in biomarkers are standardized. Primary covariates of interest are baseline step and change in standardized steps (a time-varying variable).

Abbreviations: A1M, alpha-1 microglobulin; CI, confidence interval; EGF, epidermal growth factor; IL, interleukin; KIM1, kidney injury molecule 1; MCP1, monocyte chemoattractant protein 1; NGAL, neutrophil gelatinase-associated lipcalin; SD, standard deviation; TFF3, trefoil factor 3; UMOD, uromodulin; YKL40, Chitinase-3-like protein 1.

a mg Alb/g UCr.

b mg/L per mg/dL of UCr.

c pg/mL per mg/dL of UCr.

d Higher levels of EGF and UMOD reflect improved kidney health. Lower levels of all other biomarkers reflect improved kidney health or improved systemic inflammation.

## Discussion

In this analysis of 1,381 community-dwelling sedentary older adults in the LIFE trial, we found no effect of randomization to a structured physical activity and exercise intervention on biomarkers of kidney health, including measures of glomerular injury, tubular function and repair, tubular injury, generalized inflammation, and tubulointerstitial/fibrosis. However, in observational analyses, those participants who had higher time-updated step counts showed improvements in several domains of kidney health. Specifically, participants who walked more had trajectories reflective of less glomerular injury (lower urine albumin), better tubular function (lower urine A1M and TFF3), better tubular repair (higher urine EGF and UMOD), less tubular injury (urine IL-18 and NGAL), less generalized inflammation (TNFR1 and TNFR2), and less tubulointerstitial fibrosis/repair (urine MCP1 and clusterin). In sensitivity analyses that distinguished the incremental effect of increased activity from baseline on changes in biomarkers of kidney health, we observed that changes in activity appeared most impactful in the domains of tubular injury (IL-18) and generalized inflammation (TNFR1).

Our findings are somewhat contradictory as randomization to the physical activity intervention did not result in a significant change in any kidney biomarker except for eGFR_CysC_ (as shown previously),^17^ whereas the observational analyses showed that higher time-updated step counts were associated with relative improvements in several domains of kidney health. One possible explanation for these findings is that the randomized intervention led to only modest differences in activity (291 steps/day on average) between groups. In comparison, the median and interquartile range of steps achieved by randomized participants was 1,902 (1,568 to 3,470 steps/day). In our prior study evaluating changes in eGFR, we similarly found that the observational association of physical activity with eGFR was stronger than the randomized effect, although the intervention did significantly reduce the rate of eGFR decline.^17^ The combination of these findings suggests that a larger incremental increase in exercise may be required to improve kidney tubular health. Furthermore, although exercise does appear to have beneficial effects on kidney tubular health, this does not seem to be the primary pathway to explain the effect of exercise on eGFR. The results do not suggest that eGFR improvements with physical activity are mediated by tubular injury; rather, it is possible that there is a hemodynamic component of the effect of exercise on eGFR. For example, although the effect on eGFR was independent of changes in blood pressure, the LIFE study did not measure endothelial function, which has been associated with glomerular and tubular health.^18^^,^^19^ In the general population, higher levels of physical activity correspond to better endothelial function,^20^, ^21^, ^22^ which in turn can be improved by aerobic exercise training. Furthermore, both high-intensity interval exercise and moderate-intensity exercise have been shown to improve vascular endothelial function in moderate CKD,^23^ with high-intensity exercise also transiently improving kidney filtration.^2^ Finally, it is possible that the discrepancy between results detailing the effects of the intervention on eGFR versus biomarkers of kidney health is in part related to the lower reproducibility of the biomarker assays. However, there were no significant findings for the intervention across all of the biomarkers studied, which we believe suggests that biomarker imprecision is a less likely explanation for the null results.

There are relatively few prior studies evaluating the effects of exercise on markers of systemic inflammation and/or glomerular health, and they have reported mixed results. Prior studies have differed from this LIFE ancillary study in several ways: smaller sample sizes, restriction to specific stages or etiologies of CKD, shorter exercise interventions (generally 6-12 months), and a narrower profile of kidney health measures. For example, a study of home-based aerobic exercise and resistance training for participants with stage 4 CKD showed no improvement in urine albumin over 6 months, but did show improvement in urinary liver-type fatty acid-binding protein (a marker of tubular injury) and C-reactive protein (a marker of generalized inflammation).^24^ In contrast, a 6-month trial of 121 patients with diabetic nephropathy showed that an intensive lifestyle intervention of diet control and exercise improved albuminuria over 6 months.^25^ Another 4-month intervention of diet control and aerobic exercise using a 2 × 2 factorial design in moderate-to-severe CKD showed improvements in F2-isoprostane (a marker of oxidative stress) and IL-6 (systemic inflammation) in the combined and independent interventions, but found no improvement in albuminuria.^26^ In the general population of older adults, LIFE-P (the pilot study for LIFE) assessed the effects of moderate-intensity exercise for 12 months and found improvements in serum concentrations of IL-18 but no change in other inflammatory markers, including TNFR1 and TNFR2.^27^ Therefore, it may be that the duration of activity plays a role in modifying some biomarkers of systemic inflammation in older adults. Relative to these smaller, heterogeneous studies with more limited kidney evaluation, our study measured the effects of exercise on multiple dimensions of kidney health and systemic inflammation over a 2-year intervention among a large cohort of sedentary older adults, a population at high risk of kidney function decline.

These results help to elucidate the mechanisms by which activity and exercise might affect changes across multiple dimensions of kidney health. Importantly, in addition to a reduction in glomerular filtration rate, the aging kidney has declining proximal^3^ and distal tubular function.^28^ In the present study, we found that greater time-updated step counts were associated with favorable changes in markers in both proximal (A1M, IL-18, and TFF3) and distal (NGAL) tubules, suggesting that physical activity can directly slow age-related declines in these domains. Finally, our results also support the hypothesis that exercise improves kidney health in part through its beneficial effects on systemic inflammation,^29^^,^^30^ as demonstrated by the association of higher activity with improvements in serum TNFR1 in particular and also in TNFR2 and MCP-1. The evidence is strongest for physical activity affecting domains of tubular injury and generalized inflammation because the findings are consistent in both the repeated measures analysis of the cumulative effect of exercise (Table 3) and the incremental effect of exercise (Table 4). Furthermore, our sensitivity analyses allow us to determine that the effects of exercise on biomarkers in these domains (IL-18 and TNFR1 specifically) appear to be driven by longitudinal associations and not solely from the cross-sectional associations at baseline. We believe that both sets of analyses are important and allow us to highlight the consistent directionality of all of the repeated measures analyses, in which greater activity is associated with improvements in kidney health.

It is important to note that although step counts are a measure of physical activity, this study was geared toward studying the effect of a combined-activity exercise intervention on changes in biomarkers of kidney health and not activity alone. However, the trial only included 2 measures of observed activity that could be used to evaluate a dose-response relationship between components of the intervention and changes in kidney health: step counts and time spent in moderate-intensity activity. In our prior study,^17^ we found that step counts and moderate-intensity activity were highly correlated, suggesting that most moderate-intensity activity was spent when walking. However, we cannot distinguish the potential effects of muscle-strengthening exercises on biomarkers of kidney health separately from the walking or activity component of the intervention.

There are several other limitations to be acknowledged. This is an ancillary study to an existing trial, and the original study was not designed to test the impact of physical activity and exercise on kidney health. Non-White individuals were underrepresented, and the study included few participants with advanced CKD. Furthermore, given LIFE’s focus on older individuals, these findings may not apply to a younger population. Due to the timing of specimen collection, our analyses were limited to a 2-year follow-up; we do not know whether any beneficial effects of physical activity would be increased or decreased during longer follow-up periods. There are other possible factors that can influence the ability to maintain activity as well as kidney health that this study is not able to quantify, including socio-economic factors affecting access to nutrition, transportation, or caregiving. However, the findings are both thematically and directionally consistent with the randomized effect of physical activity on improvements in eGFR, and the markers assessed here are used to understand potential mechanisms. Nonetheless, the associations of higher step counts with changes in biomarkers of kidney health are observational and are subject to residual confounding effects despite our adjustment for multiple covariates.

In summary, in a trial that employed a structured physical activity and exercise intervention for older adults, no differences in biomarkers of multiple domains of kidney health were observed between individuals randomized to structured physical activity intervention and those randomized to health education alone. However, those participants who had higher step counts had relatively improved biomarkers of kidney health, including glomerular injury, tubular function, tubular injury, generalized inflammation, and tubulointerstitial repair/fibrosis. Incremental changes from baseline in activity appeared most impactful on the domains of tubular injury and generalized inflammation. These results help to clarify the overall impact of physical activity on kidney decline. Future research should explore whether these same biomarkers of kidney health change over time as a natural course of aging and, if so, whether physical activity and exercise can slow the natural process of aging in the kidney. Additional studies are needed to evaluate which components of physical activity are most beneficial for kidney health, whether exercise intensity gives a graded benefit in kidney health, and whether persons with advanced kidney disease also benefit from physical activity.
