# Supplementary material for: Structured Moderate Exercise and Biomarkers of Kidney Health in Sedentary Older Adults: The Lifestyle Interventions and Independence for Elders Randomized Clinical Trial
Source: Kidney Med. 2023 Sep 13;5(11):100721. doi: 10.1016/j.xkme.2023.100721 (PMC10616412; doi:10.1016/j.xkme.2023.100721)
Supplement: Supplementary File (PDF) — Figure S1; Tables S1-S3. [file mmc1.pdf]

Figure S1. Flow of Participants through Clinical Trial (CONSORT)

14831 Patients assessed for eligibility

13196 Excluded
- **2654** SPPB too high
- **2422** Currently exercising too frequently
- **2321** Plan to move within 24 months
- **626** Currently mobility disabled
- **611** Morbidity exclusions
- **437** Other reasons
- **4125** Chose not to continue screening or refused

1635 Randomized

Allocation

818 Randomization to physical activity intervention
- **800** Received intervention
- **18** Did not receive intervention
  - **10** Reason unknown
  - **4** Illness/death
  - **1** Physician's advice
  - **2** Too busy
  - **1** Dissatisfied

723 Blood sample collected at baseline
- **686** with biomarkers
- **37** without biomarkers

817 Randomized to health education intervention
- **805** Received intervention
- **12** Did not receive intervention (reason unknown)

719 Blood sample collected at baseline
- **695** with biomarkers
- **24** without biomarkers

Follow-Up

142 Incomplete follow-up for biomarkers (48 year 1 and 94 year 2)
- **2** Illness
- **1** too busy
- **4** unable to contact
- **29** withdrew from study
- **22** death
- **84** unknown

140 Incomplete follow-up for eGFR (35 year 1 and 105 year 2)
- **2** Illness
- **1** caregiver responsibility
- **1** forgot appointment
- **2** unable to contact
- **1** refused to give reason
- **21** withdrew from study
- **20** death
- **92** unknown

Analysis

686 Included in analysis

695 Included in analysis

Table S1: Comparison of participants with and without biomarkers

	Without biomarkers (n=254)	*With biomarkers (n=1381)*	*Overall (n=1635)*	*p-value**
**Age, years**	79.1 (5.2)	78.8 (5.2)	78.9 (5.2)	0.45**
**Female**	191 (75.2)	907 (65.7)	1098 (67.2)	0.003
**Race**				0.78
**Black**	48 (18.9)	240 (17.4)	288 (17.6)	
**White**	188 (74.0)	1051 (76.1)	1239 (75.8)	
**Other**	18 (7.1)	90 (6.5)	108 (6.6)	
**Diabetes status**				0.009
**No Diabetes**	148 (58.3)	669 (48.4)	817 (50.0)	
**Impaired fasting glucose**	40 (15.7)	313 (22.7)	353 (21.6)	
**Diabetes**	66 (26.0)	399 (28.9)	465 (28.4)	
**CVD**	76 (29.9)	416 (30.1)	492 (30.1)	0.95
**Hypertension**	173 (69.5)	978 (71.3)	1151 (71.0)	0.56
**Total steps**	2500 (1522)	2693 (1467)	2664 (1477)	0.010***

CVD = cardiovascular disease.
*For discrete variables, chi-squared tests are used.
** t-test
*** Wilcoxon rank sum test

Table S2: Comparison of participants with and without accelerometry measures

	Without accelerometry (n=269)	***With accelerometry (n=1112)***	***Overall (n=1381)***	***p-value****
**Age, years**	79.2 (4.9)	78.8 (5.3)	78.8 (5.2)	0.19**
**Female**	181 (67.3)	726 (65.3)	907 (65.7)	0.54
**Race**				0.31
**Black**	47 (17.5)	193 (17.4)	240 (17.4)	
**White**	199 (74.0)	852 (76.6)	1051 (76.1)	
**Other**	23 (8.6)	67 (6.0)	90 (6.5)	
**Diabetes status**				0.26
**No Diabetes**	121 (45.0)	550 (49.5)	671 (48.6)	
**Impaired fasting glucose**	61 (22.7)	256 (23.0)	317 (23.0)	
**Diabetes**	87 (32.3)	306 (27.5)	393 (28.5)	
**CVD**	76 (28.3)	339 (30.5)	415 (30.1)	0.47
**Hypertension**	190 (70.9)	797 (72.0)	987 (71.8)	0.72

CVD = cardiovascular disease.
*For discrete variables, chi-squared tests are used.
** t-test

Table S3: Effect of randomization to Physical Activity (PA) intervention versus Health Education (HE) control on biomarkers of tubular function and injury at year 1 and year 2

	Year 1		Year 2	
**Biomarker (SD)**	**Standardized Change in biomarker (95% CI) when comparing PA vs. HE**	**p-value**	**Standardized Change in biomarker (95% CI) when comparing PA vs. HE**	**p-value**
**Glomerular Injury**				
Urine albumin (3.47)*	0.01 (-0.08 – 0.11)	0.76	-0.03 (-0.15 – 0.09)	0.63
**Tubular Function and Repair**				
A1M (0.54)**	0.01 (-0.04 – 0.06)	0.69	-0.01 (-0.12 – 0.11)	0.91
TFF3 (18.9)***	-0.004 (-0.09 – 0.08)	0.93	-0.10 (-0.18 – -0.02)	0.015
EGF (22.2)***	-0.04 (-0.14 – 0.06)	0.41	-0.03 (-0.14 – 0.07)	0.52
UMOD (219129)***	0.01 (-0.04 – 0.05)	0.77	-0.03 (-0.08 – 0.01)	0.11
**Tubular Injury**				
IL-18 (0.73)***	0.0006 (-0.05 – 0.05)	0.98	0.001 (-0.04 – 0.05)	0.95
NGAL (6446)***	-0.04 (-0.12 – 0.04)	0.31	-0.11 (-0.25 – 0.04)	0.16
KIM1 (urine) (16.9)***	0.07 (-0.06 – 0.19)	0.29	0.005 (-0.08 – 0.09)	0.92
KIM1 (serum) (189)	0.02 (-0.07 – 0.11)	0.69	0.07 (-0.08 – 0.21)	0.38
**Generalized Inflammation**				
TNFR1 (serum) (1090)	-0.03 (-0.13 – 0.06)	0.48	0.003 (-0.13 – 0.14)	0.96
TNFR2 (serum) (3898)	0.05 (-0.05 – 0.15)	0.35	0.11 (-0.03 – 0.24)	0.11
**Tubulointerstitial Repair/Fibrosis**				
MCP1 (3.09)***	-0.01 (-0.10 – 0.07)	0.78	-0.02 (-0.12 – 0.08)	0.69
Clusterin (6376)***	-0.01 (-0.11 – 0.09)	0.86	-0.05 (-0.17 – 0.07)	0.40
YKL40 (219.3)***	0.002 (-0.08 – 0.08)	0.97	-0.08 (-0.21 – 0.06)	0.26

Adjusted for baseline biomarker, intervention, visit, sex, clinical site, and interaction between visit and intervention)

All urine biomarkers are indexed to Cr (i.e. biomarker/Cr). Changes in biomarkers are standardized.

*mg Alb/g Cr

** mg/L per mg/dL of UCr

*** pg/mL per mg/dL of UCr

† Higher levels of EGF and UMOD reflect improved kidney health. Lower levels of all other biomarkers reflect improved kidney health or improved systemic inflammation.
